# Clinical-laboratory and ultrasound parallels of changes in the liver and thyroid gland in diffuse toxic goiter

**DOI:** 10.25122/jml-2021-0291

**Published:** 2022-01

**Authors:** Khrystyna Zynoviivna Lavruk, Petro Fedorovych Dudiy, Nadiya Vasylivna Skrypnyk, Vasyl Hryhorovych Mishchuk, Zynoviy Yaroslavovych Vytvytskiy

**Affiliations:** 1. Department of Radiology and Radiation Medicine, Ivano-Frankivsk National Medical University, Ivano-Frankivsk, Ukraine; 2. Department of Endocrinology, Ivano-Frankivsk National Medical University, Ivano-Frankivsk, Ukraine; 3. Department of General Practice (Family Medicine), Physical Rehabilitation and Sports Medicine, Ivano-Frankivsk National Medical University, Ivano-Frankivsk, Ukraine

**Keywords:** diffuse toxic goiter, ultrasound examination, shear wave elastography, DTG – diffuse toxic goiter, TG – thyroid gland, CDLD – chronic diffuse liver diseases, TSH – thyroid-stimulating hormone, T3f – free triiodothyronine, T4f – thyroxine free, ArTTG – antibodies to thyroid-stimulating hormone receptors, ALT – alanine aminotransferase, AST – aspartate aminotransferase, AP – alkaline phosphatase, SWV – shear wave velocity

## Abstract

Detection of liver dysfunction in patients with diffuse toxic goiter (DTG) at an early stage allows for correcting it in time with appropriate therapy; therefore, diagnosing hepatobiliary system lesions in these patients is an important medical issue. We examined 62 patients, divided into two groups depending on the duration of the disease. The first group included patients with a disease duration of up to two years, the second group - patients with a disease duration of more than two years. The study and comparison of laboratory and multiparametric ultrasound criteria of liver and thyroid dysfunction were performed. Analysis of ultrasound signs of hepatobiliary system lesions in patients in the two groups showed that they were more common in the second group. There is a correlation between the stiffness of the parenchyma of the thyroid gland and liver and the duration of the disease, the level of free thyroxine (T4f), and antibodies to thyroid-stimulating hormone receptors (ArTTG). Increased liver stiffness was more common in patients with ArTTG levels above 20 IU/ml, and the degree of F1 fibrosis was higher at T4f greater than 50 pmol/l. To assess the condition of a patient with DTG and the need to correct treatment tactics, it is advisable to use the criteria of multiparametric ultrasound examination of the hepatobiliary system and thyroid gland.

## Introduction

Diffuse toxic goiter (DTG, Graves’ disease) is a complex endocrinology issue, as it is often accompanied by hyperthyroidism, affecting people of working age, and being characterized by the development of complications [1]. Various organs and systems of the human body can be affected, including the cardiovascular, nervous, gastrointestinal, and hepatobiliary with hyperthyroidism against the background of DTG [2, 3]. Changes in the liver are often observed in patients with DTG; therefore, the interaction between the liver and the thyroid gland (TG) is critical for maintaining homeostasis in the body [4, 5]. Laboratory signs of damage to the digestive system are observed in a biochemical blood test in 15–76% of patients with DTG. There are several mechanisms of impaired liver function against the background of hyperthyroidism: independent effect of a large number of thyroid hormones on the liver, lesions associated with concomitant heart failure, and changes caused by the use of antithyroid drugs [6–8]. Histological examination of liver tissue in thyrotoxicosis revealed diffuse perisinusoidal and perivascular edema, hydropic hepatocyte dystrophy, atrophy of the tract, lymph histiocytic infiltration of the stroma, multiple diffuse foci of cytolysis with the formation of cavities [9]. The rapid detection of liver dysfunction can be corrected by appropriate therapy, and therefore the issue of early diagnosis of hepatobiliary system damage in patients with Graves’ disease becomes of particular importance and is an urgent medical issue.

The leading role in diagnosing chronic diffuse liver diseases (CDLD) is the intravital biopsy. However, due to invasiveness, a wide range of contraindications, and possible complications from the procedure, it remains important to study the effectiveness of additional research methods in diagnosing CDLD [10]. One of the most informative instrumental methods for diagnosing the state of the hepatobiliary system is ultrasound diagnostics in B-mode and Doppler mode. However, such an examination does not provide information on the stiffness of the liver parenchyma, which is an important diagnostic criterion in assessing its functional state and choosing treatment tactics. In recent years, much attention has been paid to the technique of ultrasound elastography – a non-invasive assessment of the elastic properties of the tissue, which allows us to assess its rigidity in response to the applied mechanical force (compression or shear wave) [11]. In various pathologies, the elasticity of tissues changes, so obtaining qualitative and quantitative information about their stiffness is of great diagnostic value in diagnosing CDLD since the development of fibrosis is a serious complication of many diseases [12]. Despite some scientific work on the use of ultrasound in liver disease, the question of sonographic changes in the hepatobiliary system in diffuse thyroid disease, the likelihood of their correlation with thyroid hormone levels, clinical history, biochemical data, and biochemical parameters remains insufficiently studied.

## Material and Methods

Multiparametric ultrasound examination of the thyroid gland and hepatobiliary system was performed on 62 patients aged 18–70 years with diffuse toxic goiter (including 55 women, 7 men). The study was conducted at St. Luke’s Medical and Diagnostic Center (Ivano-Frankivsk) during 2019–2021. Patients were divided into two groups depending on the duration of the disease. The first group included patients with newly detected diffuse toxic goiter and a disease duration of up to two years (32 patients – 52%); the second group included patients with a disease duration of more than two years (30 patients – 48%). The control group included 30 people without signs of thyroid damage, which was confirmed by laboratory and ultrasound examinations. The following clinical characteristics were established in patients: irritability, bulging eyes, weight loss, palpitations, sweating.

Assessment of thyroid gland function was performed based on the results of a blood test to determine the thyroid panel, namely: thyroid-stimulating hormone (TSH), free triiodothyronine (T3f), and thyroxine (T4f), antibodies to thyroid-stimulating hormone receptors (ArTTG).

Liver status was assessed by the level of alanine aminotransferase (ALT), aspartate aminotransferase (AST), total bilirubin, alkaline phosphatase (AP) in the biochemical analysis of blood. The following level of indicators was considered as the norm: TTG – 0.35–5.1 mMo/l, T3f – 3.69–6.45 pmol/l, T4f – 12.0–22.0 pmol/l, ArTTG – to 1.75 Units/l, ALT – 7.0–41.0 U/l, AST – 10.0–40.0 U/l, total bilirubin – 3.4–18.8 μmol/l, LF – 36–105 U/l. Examinations were performed at the initial treatment of the patient. Patients were prescribed therapy with tyrosol. Dosage and duration of treatment were determined depending on the dynamics of changes in thyroid hormone levels. The course of treatment was mostly prescribed for 12–18 months, and other tactics depended on the clinical case. The daily dose of tyrosol ranged from 10 mg to 20 mg, maintenance – 5 mg. All patients underwent ultrasound examinations of the thyroid gland and liver, performed by a radiologist with more than five years of experience. Ultrasound examinations were performed on Siemens Acuson S3000 and S2000 devices. Examination of the thyroid gland was performed using a linear sensor with a frequency of 9 MHz in B-mode, color and pulse-wave Doppler sonography modes, and shear-wave elastography. A two-dimensional scan of the thyroid gland determined its total volume in cm^3^, echogenicity, and echostructure of the parenchyma. Blood supply was evaluated using color Doppler imaging. Signs of thyroid pathology were an increase in its total volume, increased blood flow, heterogeneity of the echostructure, and a decrease in the echogenicity of the parenchyma. Shear wave elastography was performed to determine the shear wave velocity in m/s at five parenchymal points, followed by determining the mean. The stiffness of the thyroid parenchyma was considered to be increased with an increase in the average shear wave velocity (SWV) above 2.54 m/s [13].

Multiparametric ultrasound examination of the liver was performed using a convex sensor with a frequency of 6 MHz in B-mode, color and pulse-wave Doppler sonography modes, and shear-wave elastography. The examination was performed with the patient “lying on his/her back” position with free breathing, the right hand located behind the head. In the B-mode, the size of the right and left lobes of the liver (within the age norm, increased), echogenicity (isoechogenic, hypoechoic, hyperechoic) and echostructure (homogeneous, heterogeneous), the presence of sediment in the gallbladder, the state of the intrahepatic ducts, and the size of the spleen were evaluated. The portal vein was evaluated in B-mode and color Doppler sonography with measurement of its diameter around the liver gate in mm (normally up to 13 mm), after which the blood flow rate in it was determined in cm/s (normal values 16–40 cm/s) and the type of blood flow (normally hepatopetal). Examination of the inferior vena cava was performed with incomplete breath retention on inspiration with the determination of the type of blood flow (phase, antegrade) and its diameter (normally – up to 20 mm). In the pulse-wave Doppler sonography, the resistance index of the hepatic artery was determined (normally 0.55–0.7). Ultrasound signs of changes in the hepatobiliary system in B-mode were constant in the presence of the following parameters: an increase in the size of the right (more than 14 cm) and left (more than 6 cm) liver lobes, an increase in echogenicity, and heterogeneity of the parenchyma, the diameter of the portal vein more than 13 mm and the inferior vena cava more than 20 mm, the presence of sediment in the gallbladder, an increase in the diameter of the choledoch (more than 6 mm), compaction of the intrahepatic bile ducts, an increase in the size of the spleen (longitudinal size more than 12 cm and transverse size more than 6 cm), growth of the diameter of the splenic vein (more than 7 mm), the presence of free fluid. When performing color and pulse-wave Doppler mapping, changes in blood supply were established at the rate of blood flow in the portal vein below 16 cm/s, the appearance of hepatofugal type of blood flow in the portal vein, and pulsating type of blood flow in the inferior vena cava, an increase in the resistance index in the hepatic artery above 0.7 [14–16].

Shear-wave elastography of the liver was performed using a curvilinear transducer using intercostal access, with the central beam positioned perpendicular to the liver capsule. Measurements of the liver parenchyma stiffness in kPa were performed in the VirtualTouch program, the area of interest was established in areas without large vessels and far from the gallbladder. Indicators were determined at ten points of the right lobe at a depth of 3–8 cm from the skin surface, followed by a determination of the average indicator. Determination of the degree of liver fibrosis was carried out according to the METAVIR scale, according to which the following liver stiffness indicators corresponded to the degrees of fibrosis: F0 (no fibrosis) ≤6.0 kPa, F1 (mild fibrosis) – 6.0 to 7.0 kPa, F2 (moderate fibrosis) – 7.0 to 9.5 kPa, F3 (severe fibrosis) – 9.5 to 12.5 kPa, F4 (severe fibrosis) >12.5 kPa [10, 17, 18].

This study is cross-sectional. The criteria for excluding patients from the study were: viral hepatitis B and C, alcohol dependence, autoimmune hepatitis, hepatotoxic effects of substances associated with occupational diseases, pregnancy, radioactive iodine therapy, and thyroid surgery.

The Stat Soft Statistical software, Version 6.0, was used to process statistical data. Quantitative changes were expressed as mean±SD, qualitative changes as a percentage. The difference between the two groups was determined using Student’s t-test. P value<0.05 was considered statistically significant.

## Results

The characteristics of gender, age, duration of the disease, and antithyroid medication intake in 62 patients in both groups are presented in Table 1. The findings showed that both groups were dominated by female patients, but the quantitative composition of the males in the second group was higher than in the first one. The age composition of both groups was similar.

**Table 1.** Characteristics of groups of examined patients.

**Characteristics**	**Group 1 (n=32)**	**Group 2 (n=30)**
**Age (years)**	45±12.8	48±13.2
**Male**	2	5
**Female**	30	25
**Disease duration (months)**	12±11.5	98±73.8
**Duration of taking antithyroid medications (months)**	9±4.2	41±29.2

According to laboratory studies of the thyroid panel, the following changes were found: a decrease in the level of thyroid-stimulating hormone was detected in 32 (100%) patients from group 1 and 26 (87%) from group 2, an increase in free thyroxine, respectively, in 32 (100%) and 25 (83%), triiodothyronine – in 29 (91%) and 25 (83%), antibodies to thyroid-stimulating hormone receptors – in 32 (100%) and 23 (77%) patients, respectively. An increase in ALT levels was observed in 25 (78%) patients of the first and 18 (60%) of the second group, an increase in the concentration of drugs was observed in 15 (47%) and 9 patients (30%), respectively, total bilirubin, 11 (34%) and 4 patients (13%), respectively. However, patients in group 2 (10 patients, 33%) were more likely to have an increase in AST levels than those examined in the first group (7 patients, 22%). Of all the examined patients in both groups, patients with abnormal values of the indicators of the functional state of the liver and thyroid panel were singled out (Table 2). According to laboratory tests, patients in the first group had a more pronounced decrease in thyroid-stimulating hormone and increased T4f, T3f, and ArTTG levels. Clinical symptoms of the disease were more common in patients from the first group than in the second. In percentage terms, irritability was found in 91% and 80% of patients, respectively, bulging eyes – in 56% and 32%, weight loss – in 76% and 57%, palpitations – in 93% and 84%, sweating – in 78% and 58%. There was a positive correlation between thyroid hormone levels and the presence of clinical symptoms.

**Table 2.** Results of laboratory examinations of the functional state of the liver and thyroid panel.

**Laboratory indicator**	**Group 1 (n=32)**		**Group 2 (n=30)**	
	**Indicator level**	**Percentage of the number of patients in the group with abnormal indicators**	**Indicator level**	**Percentage of the number of patients in the group with abnormal indicators**
**TSH (mMo/l)**	0.03±0.013	100%	0.18±0.05	87%
**T4 f (pmol/L)**	51.1±18.1	100%	40.5±16.3	83%
**T3 f (pmol/l)**	28.2±10.2	91%	21.6±12.1	83%
**ArTTG (IU/ml)**	23.4±10.7	100%	21.1±11.5	77%
**Total bilirubin (mmol/l)**	31.1±6.2	34%	25.9±5.5	4%
**ALT (U/l)**	83.0±23.0	25%	61.0±15.0	18%
**AST (U/l)**	52.0±10.0	22%	57.0±8.0	33%
**Alkaline phosphatase (IU/ml)**	189±18.9	47%	163±17.1	30%

The indicators obtained by B-mode ultrasound and Doppler imaging of the thyroid gland and hepatobiliary system are presented in Tables 3 and 4, respectively.

**Table 3.** Results of ultrasound examination of the thyroid gland.

**Indicator**	**Group 1 (n=32)**		**Group 2 (n=30)**	
	**Number of patients**	**Percentage ratio**	**Number of patients**	**Percentage ratio**
**Increase in the total TG volume**	26	81%	22	73%
**Increased blood flow in TG**	31	97%	26	87%
**Inhomogeneous TG echostructure**	29	90%	30	100%
**Reduced TG echogenicity**	26	81%	27	90%
**Increase in average rigidity of TG parenchyma**	18	56%	26	87%

**Table 4.** Results of ultrasound examination of the hepatobiliary system.

**Indicator**	**Group 1 (n=32)**		**Group 2 (n=30)**	
	**Number of patients**	**Percentage ratio**	**Number of patients**	**Percentage ratio**
**Increased oblique vertical size of the liver right lobe**	12	37%	17	56%
**Increased thickness of the liver left lobe**	8	25%	15	50%
**Increased echogenicity of the liver parenchyma**	6	19%	13	24%
**Heterogeneous echostructure of the liver parenchyma**	1	29%	10	33%
**Contents of the gallbladder with sediment**	22	69%	29	97%
**Condensed intrahepatic bile ducts**	9	28%	15	50%
**Portal vein diameter, more than 13 mm**	2	6%	12	40%
**The rate of blood flow in the portal vein is lower 16 cm/s**	0	-	6	20%
**Inferior vena cava diameter more 20 mm**	0	-	11	16%
**Pulsating blood flow in the inferior vena cava**	0	-	4	13%
**Hepatic artery resistance Index – greater than 0.7**	0	-	4	13%
**Increased spleen size**	5	15%	8	26%
**Spleen vein diameter – more than 13 mm**	2	6%	5	16%

**Figure 1.**
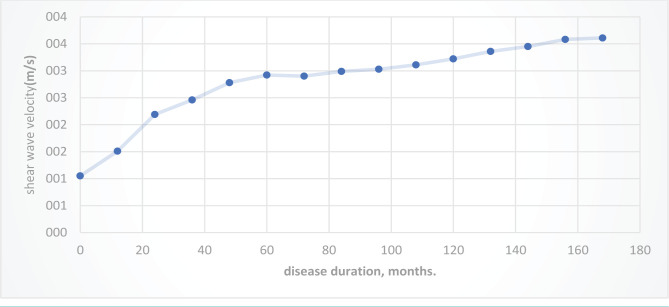
Shear wave velocity (m/s) passing through the TG parenchyma in patients of both groups, depending on the duration of the disease.

Comparison of the ultrasound picture of thyroid changes in patients of the two groups showed that an increase in its volume is observed more often in patients of the first, and ultrasound signs of diffuse parenchymal changes are more pronounced in patients of the second. In group 2, an increase in SWV was more often observed when passing through the TG parenchyma above 2.54 m/s. Average SWV values in patients from group 1 amounted to 1.53±0.48 m/s, and in the second group, 2.9±0.71 m/s. There was also an increase in the disease duration, which is reflected in Figure 1.

**Figure 2.**
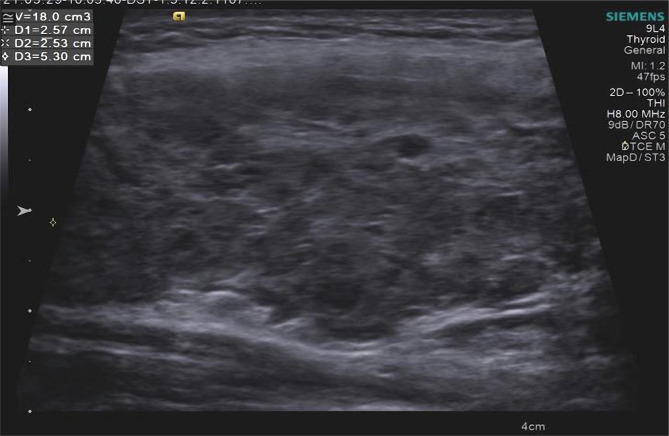
TG sonogram of patient K., 45, with DTG. Heterogeneity of the parenchyma echostructure due to areas of reduced and increased echogenicity.

**Figure 3.**
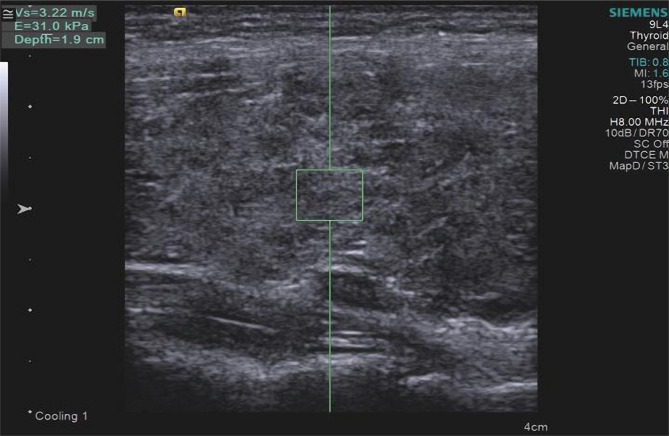
TG sonoelastography of patient K., 45, with DTG. Increased SWV when passing through the parenchyma.

For example, the results of ultrasound examination of the thyroid gland in DTG in patient K., 45 years old, are presented:

• Heterogeneity of the thyroid parenchyma in B-mode is shown in Figure 2;
• SWV growth greater than 2.54 m/s during elastography is shown in Figure 3.

Analysis of changes in ultrasound signs of damage to the hepatobiliary system of patients in two groups showed that all the deviations mentioned above were more often observed in group 2. According to the ultrasound picture, a small number of patients in the first group had changes such as an increase in liver size, increased echogenicity, heterogeneity of the echostructure, and an enlarged spleen. These changes were observed with an even higher frequency in patients from group 2. Other indicators in the B-mode in patients from group 1 were within the normal range, while in those examined in group 2, dilation of the portal (40%) and inferior vena cava (16%) was observed. During Doppler imaging, patients in group 1 did not show any abnormalities in the blood flow of liver vessels, while some patients in group 2 showed an increase in the speed of blood flow in the portal vein, an increase in the resistance index in the hepatic artery, and the appearance of pulsating blood flow in the inferior vena cava. The results of the liver examination in shear-wave elastography are presented in Table 5.

**Table 5.** Results of shear wave elastography of the liver.

**Degree of fibrosis**	**Group 1 (n=32)**		**Group 2 (n=30)**	
	**Number of patients**	**Percentage ratio**	**Number of patients**	**Percentage ratio**
**F0**	24	75%	8	27%
**F1**	6	19%	11	37%
**F2**	2	6%	6	20%
**F3**	-	-	3	10%
**F4**	-	-	2	6%

**Figure 4.**
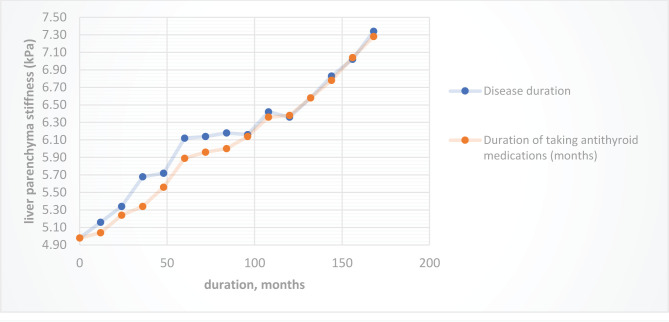
Indicators of liver parenchymal stiffness (kPa) in patients of both groups, depending on the duration of the disease and the duration of taking antithyroid drugs.

**Figure 5.**
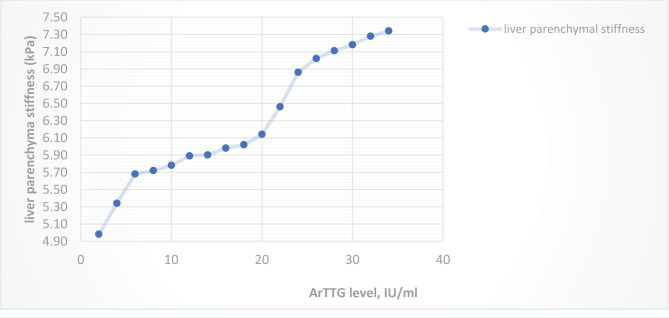
Indicators of liver parenchymal stiffness (kPa) in patients of both groups depending on the level of ArTTG (IU/ml).

Also, patients in group 1 mainly showed fibrosis of the F1 degree, and patients in group 2 – F1, F2, F3, F4 degrees. In 24 patients in group 1 with F0 degree of fibrosis, the average liver stiffness was 4.56±1.17 kPa, in 6 patients with F1 fibrosis, this indicator was 6.62±0.49 kPa, in 2 patients with F2 fibrosis, the stiffness was equal to 8.5kPa and 8.9 kPa. In patients from the second group with a degree of fibrosis F0, the average liver stiffness was 5.2±0.51 kPa, with F1 – 6.6±0.56 kPa, with F2 – 8.5±0.64 kPa, with F3 – 10.1±0.46 kPa. Patients in group 2, whose degree of fibrosis corresponded to F4, had liver stiffness indicators of 12.62 kPa and 12.98 kPa. Increased liver stiffness is positively correlated with an increase in the duration of the disease and the use of antithyroid drugs (Figure 4), the level of ArTTG in the blood (Figure 5).

The degree of F1 fibrosis in group 1 was observed more often in the presence of high levels of T4f (above 50 pmol/l). In patients from the two groups whose ArTTG levels exceeded 20 IU/ml, increased liver stiffness was more often observed.

Relapses of hyperthyroidism after tyrosol withdrawal were observed in 25 patients from the second group. Of these, increased stiffness of the liver parenchyma was observed in 20 patients (80%), with a degree of fibrosis F1 detected in 10 patients, F2 – in 5, F3 – in 3, F4 – in 2. The level of ArTTG was above 20 IU/ml in 14 patients (56%) with recurrent hyperthyroidism.

**Figure 6.**
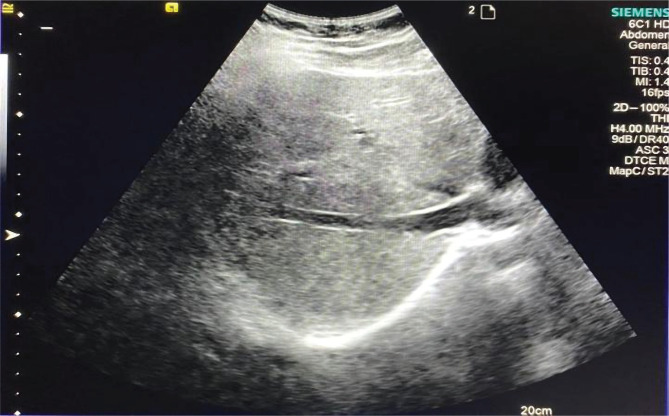
Sonogram of the liver in patient M., 56, with DTG. Heterogeneity of the parenchyma echostructure.

**Figure 7.**
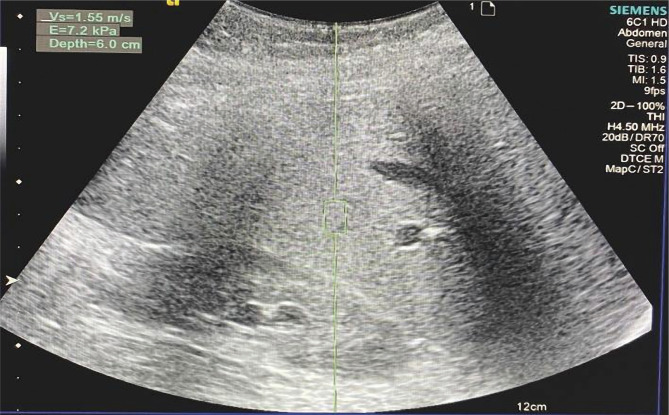
Sonoelastogram of the liver in patient M., 56, with DTG. Increased stiffness of the liver parenchyma, an indicator of 7.2 kPa, corresponds to the degree of fibrosis F2.

As an example, the results of ultrasound examination of patient M., 56, from group 2, whose ArTTG level was 34 IU/ml, are presented:

• The heterogeneity of the liver parenchyma in the B-mode is shown in Figure 6;
• Increase in the stiffness of the liver parenchyma, which corresponds to the degree of fibrosis F2, is shown in Figure 7.

## Discussion

Laboratory and ultrasound signs of liver dysfunction were studied and compared depending on the duration of the disease in patients with diffuse toxic goiter. The results of studying disorders of the hepatobiliary system in patients with hyperthyroidism, described in various sources, are heterogeneous. There is also no consensus on the pathogenesis of liver damage in this disease and the degree of changes in it depending on the duration of the disease, the level of thyroid hormones, and the duration of treatment with antithyroid drugs. Lorenzo Scappaticcio, Miriam Longo [19] indicate a frequent increase in liver samples in patients with newly diagnosed and untreated hyperthyroidism. They indicated that the frequency of deviations of liver samples in this category of patients was ALT 83%, AST – 87%, LF – 53%, total bilirubin – 50%, and after antithyroid therapy, ALT normalized in 83%, AST in 87%, LF in 53% of patients. In our study, patients in the first group also showed an increase in ALT levels in 79%, ALP in 48%, total bilirubin in 43%, which is consistent with the data of Lorenzo Scappaticcio, Miriam Longo, but the AST level in these patients, according to our data, increased in 63%, which is lower than in the results described above.

In patients from group 2, an increase in liver samples was observed less frequently, which may be explained by a lower effect on hepatocytes of an increased level of thyroid hormones because the level of T4f in group 1 was equal to 51.1±18.1 pmol/l *vs.* 40.5±16.3 pmol/l in group 2, T3f, respectively, 28.2±10.2 pmol/l and 21.6±12.1 pmol/l. According to the ultrasound picture, echographic changes in the hepatobiliary system in group 1 were observed less frequently than in group 2, which may be associated with deeper changes in the structure of the liver and heart failure. In our opinion, these changes are caused to a greater extent by the influence of prolonged use of antithyroid drugs, frequent increase in thyroid hormones in the recurrent course of the disease, and the appearance of heart failure and pulmonary hypertension. Doppler changes in the liver vessels were observed only in patients from group 2 with a degree of fibrosis F2, F3. A correlation was found between the duration of diffuse toxic goiter, antithyroid therapy, and ultrasound changes in the liver, indicating the need to consider these criteria when choosing treatment tactics. The stiffness of the liver parenchyma increased in 80% of patients with recurrent hyperthyroidism, which suggests a relationship between them. Increased liver and thyroid stiffness were more common in patients with ArTTG levels above 20 and high levels of free thyroxine. In the study conducted by He K *et al.*, [20], it was shown that patients with DTG who had high ArTTG levels were more likely to develop biochemical abnormalities of the hepatobiliary system, which is consistent with our study, which established a positive correlation between the level of these antibodies and the stiffness of the liver parenchyma. A correlation was established between the stiffness of the thyroid parenchyma and the duration of the disease, the level of T4f and ArTTG is consonant with the data of Shimei Li *et al.*, [21], who also noted that the duration of the disease, Arttg levels, thyroid size, and Isthmus thickness positively correlated with its stiffness in DTG. Based on the analysis of the correspondence of changes in the liver and TG at DTG, it can be concluded that their mutual direct dependence.

## Conclusion

A study of patients in the two groups showed that an increase of liver samples was more common in the short course of the disease, although ultrasound showed no significant changes. It was found that structural changes in the liver were more often observed with prolonged illness and taking antithyroid drugs for more than two years, recurrent hyperthyroidism, high titers of ArTTG (above 20 IU/ml) and T4f (50 pmol/l), with thyroid stiffness above 2.54 m/s.

Ultrasound signs of liver damage are manifested in cases of pronounced diffuse changes. To assess the condition of a patient with DTG and the need to correct treatment tactics, it is advisable to apply such criteria as an increase in the size of the liver and spleen, an increase in echogenicity and the appearance of heterogeneity of the parenchyma echostructure, an expansion of the diameter of the portal and inferior vena cava, an increase in the blood flow rate in the portal vein, an increase in the resistance index in the hepatic artery and the appearance of pulsating blood flow in the inferior vena cava, an increase in stiffness to the degree of F1-F4, an increase in thyroid stiffness.

## Acknowledgements

### Conflict of interest

The authors declare no conflict of interest.

### Ethical approval

Ethical approval was obtained from the Ethical Commission of Ivano-Frankivsk National Medical University (approval no. 110/19-2019).

### Consent to participate

All participants agreed to participate in the study and gave written informed consent for participation in this study.

### Funding

This study was funded by Ivano-Frankivsk National Medical University, Ivano-Frankivsk, Ukraine.

### Authorship

KZL contributed to conceptualizing and data analysis, NVS contributed to the methodology. PFD contributed to writing the original draft, VHM contributed to editing the manuscript. ZYV contributed to data collection.
